# Prognostic Factors of New-Onset Hypertension in New and Traditional Hypertension Definition in a Large Taiwanese Population Follow-up Study

**DOI:** 10.3390/ijerph192416525

**Published:** 2022-12-09

**Authors:** Yi-Hsueh Liu, Szu-Chia Chen, Wen-Hsien Lee, Ying-Chih Chen, Po-Chao Hsu, Wei-Chung Tsai, Chee-Siong Lee, Tsung-Hsien Lin, Chih-Hsing Hung, Chao-Hung Kuo, Ho-Ming Su

**Affiliations:** 1 Graduate Institute of Clinical Medicine, College of Medicine, Kaohsiung Medical University, Kaohsiung 80708, Taiwan; 2 Department of Internal Medicine, Kaohsiung Municipal Siaogang Hospital, Kaohsiung 80708, Taiwan; 3 Division of Cardiology, Department of Internal Medicine, Kaohsiung Medical University Hospital, Kaohsiung 80756, Taiwan; 4 Faculty of Medicine, College of Medicine, Kaohsiung Medical University, Kaohsiung 80708, Taiwan

**Keywords:** hypertension definition, new-onset hypertension, Taiwan Biobank

## Abstract

The aim of this study was to determine the predictors of new-onset hypertension when the definition of hypertension is changed from the traditional definition (140/90 mmHg) to a new definition (130/80 mmHg). Using data from the Taiwan Biobank, a total of 17,072 and 21,293 participants in the new and traditional definition groups were analyzed, respectively. During a mean follow-up period of 3.9 years, 3641 and 3002 participants developed hypertension in the new and traditional definition groups, respectively. After multivariable analysis, older age (OR, 1.035; 95% CI, 1.030 to 1.039; *p* < 0.001), male sex (OR, 1.332; 95% CI, 1.194 to 1.486; *p* < 0.001), high systolic blood pressure (SBP) (OR, 1.067; 95% CI, 1.062 to 1.073; *p* < 0.001), high diastolic blood pressure (DBP) (OR, 1.048; 95% CI, 1.040 to 1.056; *p* < 0.001), high heart rate (OR, 1.007; 95% CI, 1.002 to 1.012; *p* = 0.004), high body mass index (BMI) (OR, 1.091; 95% CI, 1.077 to 1.106; *p* < 0.001), high fasting glucose (OR, 1.004; 95% CI, 1.001 to 1.006; *p* = 0.002), and high triglycerides (OR, 1.001; 95% CI, 1.000 to 1.001; *p* = 0.004) were significantly associated with new-onset hypertension in the new definition group. In the traditional definition group, the predictors of new-onset hypertension were older age (OR, 1.038; 95% CI, 1.032 to 1.043; *p* < 0.001), high SBP (OR, 1.078; 95% CI, 1.072 to 1.084; *p* < 0.001), high DBP (OR, 1.039; 95% CI, 1.031 to 1.046; *p* < 0.001), high heart rate (OR, 1.005; 95% CI, 1.000 to 1.010; *p* = 0.032), high BMI (OR, 1.072; 95% CI, 1.058 to 1.087; *p* < 0.001), high fasting glucose (OR, 1.003; 95% CI, 1.000 to 1.005; *p* = 0.020), low cholesterol (OR, 0.998; 95% CI, 0.997 to 0.999; *p* = 0.004), high triglycerides (OR, 1.001; 95% CI, 1.000 to 1.001; *p* = 0.001), and low estimated glomerular filtration rate (eGFR) (OR, 0.995; 95% CI, 0.993 to 0.997; *p* < 0.001). In conclusion, older age, high SBP and DBP, high heart rate, high BMI, high fasting glucose, and high triglycerides were useful predictors of new-onset hypertension in both the new and traditional definition groups. However, male sex was a significant predictor of new-onset hypertension only in the new definition group, and low cholesterol and low eGFR were significant predictors of new-onset hypertension only in the traditional definition group. Hence, changing the diagnostic cut-off value for hypertension may have a significant impact on the association of some clinical and laboratory parameters with new-onset hypertension.

## 1. Introduction

Hypertension is a common disease affecting around 20–30% of people worldwide [1], and it is an important risk factor for cardiovascular disease [2], cerebrovascular disease [3], and chronic kidney disease [4]. Hypertension is associated with a mortality rate of around 15%, and it has become a significant health burden globally [5]. Moreover, hypertension may be the most important modifiable cause of cardiovascular disease, and all-cause mortality worldwide [6]. Because of the high prevalence and global burden, prevention and control strategies for hypertension are an important public health issue, and the early identification of high-risk individuals who can benefit from preventive strategies has become a vital part of hypertension management.

Since the release of the Fifth Report of the Joint National Committee on high blood pressure in 1993 [7], the diagnostic cut-off value of hypertension has remained at 140/90 mmHg for the past 30 years. However, the SPRINT and STEP trials provided sufficient evidence to change this 140/90 mmHg cut-off value [8,9]. In these two trials, lowering systolic blood pressure (SBP) intensively to <130 mmHg compared to the traditional SBP target of <140 (130–139) mmHg consistently reduced the relative risk of cardiovascular events by 25–30%. Therefore, a lower threshold (≥130/80 mmHg) for defining hypertension is recommended in several hypertension guidelines [10,11]. 

Several risk prediction models for incident hypertension have been developed [12] using predictors including age, body mass index (BMI), systolic blood pressure (SBP), and diastolic blood pressure (DBP), all of which are easily obtained in clinical practice. A few studies have also considered blood biochemistry factors or anthropometric parameters [13,14] as predictors. The Chin-Shan Community Cardiovascular Cohort Study also proposed a prediction model for the risk of new-onset hypertension in ethnic Chinese in Taiwan [15]. Since hypertension is also considered to be a metabolic disease, changes in blood biochemical factors could provide important and valuable information for predicting hypertension. 

Nearly all hypertension studies investigating the risk factors for future hypertension development have used the traditional cut-off value of 140/90 mmHg to define hypertension [16,17], and very few studies have used the new diagnostic cut-off value of 130/80 mmHg. Therefore, the aims of this study were to determine the significant predictors of future hypertension development when using the new definition of hypertension (130/80 mmHg), and to evaluate the impact on predictors of new-onset hypertension when the diagnostic cut-off value is changed from 140/90 mmHg to 130/80 mmHg. 

## 2. Methods

### 2.1. Study Participants

The Taiwan Biobank (TWB) is sponsored by the government of Taiwan and collects data on the genetic and lifestyle factors of Taiwanese residents who are 30–70 years of age with no history of cancer [18,19]. All participants in the TWB undergo physical examinations, provide blood samples, and complete questionnaires to obtain data on personal information and medical history, including diabetes mellitus (DM) and hypertension.

**Figure 1:**
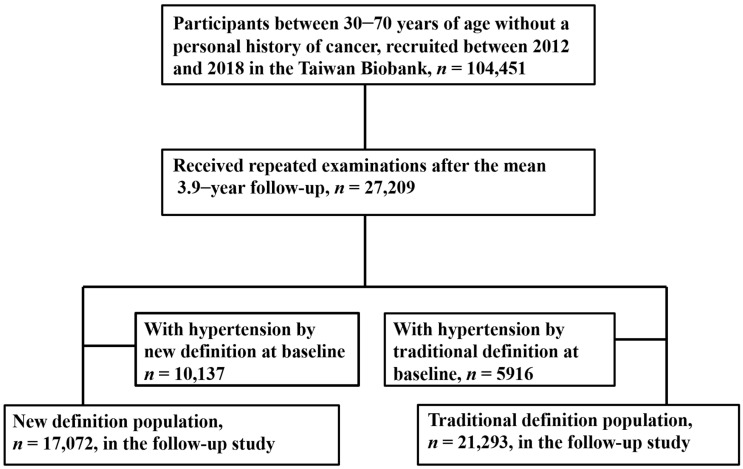
Flowchart of study population.

From 2012 to 2018, 104,451 people were registered in the TWB. Personal and medical data including age, sex, smoking history, BMI, SBP, DBP, and resting heart rate were collected. Blood chemistry data including serum creatinine, glucose, triglycerides, and total cholesterol were also collected after an overnight fast. Estimated glomerular filtration rate (eGFR) was calculated using the four-variable MDRD equation [20]. Because this study was designed to evaluate the determinants of future hypertension development, we only included participants with complete data at baseline and after 3.9 years of follow-up (*n* = 27,209). We excluded those with hypertension at baseline (*n* = 10,137, if the hypertension diagnostic cut-off value was 130/80 mmHg; *n* = 5916, if the hypertension diagnostic cut-off value was 140/90 mmHg). Hence, we had two study populations, the new definition population (cut-off value 130/80 mmHg) and traditional definition population (cut-off value 140/90 mmHg). We then divided the study participants according to who did (with the new-onset hypertension group) and did not (without the new-onset hypertension group) develop new-onset hypertension during follow-up. As shown in Figure 1, 17,072 and 21,293 participants in the new and traditional definition groups were entered into the analysis, respectively.

### 2.2. Definition of New-Onset Hypertension

Participants who did not receive anti-hypertensive drugs, those with no prior hypertension history, those with SBP/DBP < 130/80 mmHg in the new definition population, and those with SBP/DBP < 140/90 mmHg in the traditional definition population were defined as not having hypertension. New-onset hypertension was defined as those who were subsequently diagnosed with hypertension during follow-up.

### 2.3. Ethics Statement

Informed consent was obtained from all enrollees in the TWB. The Institutional Review Board (IRB) of Kaohsiung Medical University Hospital approved this study (KMUHIRB-E(I)-20210058). Ethical approval for the TWB was granted by the IRB on Biomedical Science Research, Academia Sinica, Taiwan, and the Ethics and Governance Council of the TWB. In addition, the study was conducted according to the Declaration of Helsinki. 

### 2.4. Statistical Analysis

Data were expressed as mean ± standard deviation for continuous variables and percentages for categorical variables. Continuous variables were compared between groups using the independent samples *t*-test. Categorical variables were compared between groups using the chi-square test. Associations between new-onset hypertension and the studied variables were analyzed using univariable and multivariable binary logistic regression analyses. Statistically significant variables in the univariable analysis were selected into the multivariable analysis; *p* values < 0.05 were considered significant. Furthermore, SPSS version 22.0 (IBM, Armonk, NY, USA) was used for all analyses.

## 3. Results

### 3.1. Comparisons of Baseline Characteristics between the with and without New-Onset Hypertension Groups

During the follow-up period, 3641 (21.3%) of the 17,072 subjects in the new definition population developed new-onset hypertension and 13,431 (78.7%) did not. The baseline characteristics of the two groups are shown in Table 1. The with new-onset hypertension group had a higher proportion of males, an older age, more smokers, a higher rate of DM, higher heart rate, DBP, SBP, BMI, triglycerides, total cholesterol and fasting glucose, and a lower eGFR than the without new-onset hypertension group. In addition, the male participants had a higher baseline SBP (111 ± 9 vs. 106 ± 11 mmHg, *p* < 0.001) and DBP (70 ± 6 vs. 66 ± 7 mmHg, *p* < 0.001) than the female participants in the new definition population. The longitudinal changes in SBP (9.8 ± 12.4 vs. 7.7 ± 12.6 mmHg, *p* < 0.001) and DBP (3.5 ± 8.2 vs. 2.6 ± 8.1 mmHg, *p* < 0.001) in the male participants were also higher than those in the female participants in the new definition population. 

In addition, 3002 (14.1%) of the 21,293 subjects in the traditional definition population developed new-onset hypertension and 18,291 (85.9%) did not. The baseline characteristics of the two groups are shown in Table 2. The with new-onset hypertension group had a higher proportion of males, an older age, more smokers, a higher rate of DM, higher heart rate, DBP, SBP, BMI, triglycerides, total cholesterol and fasting glucose, and a lower eGFR than the without new-onset hypertension group. In addition, the male participants had a higher baseline SBP (115 ± 11 vs. 110 ± 13 mmHg, *p* < 0.001) and DBP (73 ± 8 vs. 68 ± 9 mmHg, *p* < 0.001) than the female participants in the traditional definition population. The longitudinal changes in SBP (9.0 ± 13.0 vs. 7.3 ± 13.1 mmHg, *p* < 0.001) and DBP (2.4 ± 8.6 vs. 2.0 ± 8.6 mmHg, *p* = 0.002) in the male participants were also higher than those in the female participants in the traditional definition population.

### 3.2. Factors Associated with New-Onset Hypertension in the New and Traditional Definition Populations

Associations between the studied variables and new-onset hypertension in the new definition population (*n* = 17,072) were analyzed using univariable and multivariable analyses (Table 3). Univariable analysis showed that older age, male sex, smoking history, DM, high SBP, high DBP, high heart rate, high BMI, high fasting glucose, high total cholesterol, high triglycerides, and low eGFR were associated with new-onset hypertension. After multivariable analysis, older age (OR, 1.035; 95% CI, 1.030 to 1.039; *p* < 0.001), male sex (OR, 1.332; 95% CI, 1.194 to 1.486; *p* < 0.001), high SBP (OR, 1.067; 95% CI, 1.062 to 1.073; *p* < 0.001), high DBP (OR, 1.048; 95% CI, 1.040 to 1.056; *p* < 0.001), high heart rate (OR, 1.007; 95% CI, 1.002 to 1.012; *p* = 0.004), high BMI (OR, 1.091; 95% CI, 1.077 to 1.106; *p* < 0.001), high fasting glucose (OR, 1.004; 95% CI, 1.001 to 1.006; *p* = 0.002), and high triglycerides (OR, 1.001; 95% CI, 1.000 to 1.001; *p* = 0.004) were significantly associated with new-onset hypertension.

Associations between the studied variables and new-onset hypertension in the traditional definition population (*n* = 21,293) were analyzed using univariable and multivariable analyses (Table 4). Univariable analysis showed that older age, male sex, smoking history, DM, high SBP, high DBP, high heart rate, high BMI, high fasting glucose, high total cholesterol, high triglycerides, and low eGFR were associated with new-onset hypertension. Multivariable analysis showed that new-onset hypertension was significantly associated with older age (OR, 1.038; 95% CI, 1.032 to 1.043; *p* < 0.001), high SBP (OR, 1.078; 95% CI, 1.072 to 1.084; *p* < 0.001), high DBP (OR, 1.039; 95% CI, 1.031 to 1.046; *p* < 0.001), high heart rate (OR, 1.005; 95% CI, 1.000 to 1.010; *p* = 0.032), high BMI (OR, 1.072; 95% CI, 1.058 to 1.087; *p* < 0.001), high fasting glucose (OR, 1.003; 95% CI, 1.000 to 1.005; *p* = 0.020), low cholesterol (OR, 0.998; 95% CI, 0.997 to 0.999; *p* = 0.004), high triglycerides (OR, 1.001; 95% CI, 1.000 to 1.001; *p* = 0.001), and low eGFR (OR, 0.995; 95% CI, 0.993 to 0.997; *p* < 0.001).

We also investigated the determinants of follow-up SBP, DBP, pulse pressure (PP) and mean arterial pressure (MAP) in the new and traditional definition populations. For follow-up SBP, age, sex, smoking history, BMI, fasting glucose, total cholesterol, and triglycerides were useful predictors in both the new and traditional definition populations (Tables S1 and S2). However, eGFR was a significant predictor of follow-up SBP only in the new definition group, and DM was a significant predictor of follow-up SBP only in the traditional definition group. For follow-up DBP, age, sex, heart rate, BMI, fasting glucose, total cholesterol, and triglycerides were useful predictors in both the new and traditional definition populations (Tables S3 and S4). However, eGFR was a significant predictor of follow-up DBP only in the traditional definition population. For follow-up PP, age, sex, DM, heart rate, BMI, fasting glucose, and triglycerides were useful predictors in both the new and traditional definition populations (Tables S5 and S6). However, total cholesterol and eGFR were significant predictors of follow-up PP only in the traditional definition population. For follow-up MAP, the predictors were the same in both the new and traditional definition populations, including age, sex, DM, heart rate, BMI, fasting glucose, total cholesterol, and triglycerides (Tables S7 and S8).

### 3.3. Discussion

In this study, we investigated the significant determinants of new-onset hypertension when the new definition of hypertension (130/80 mmHg) was used, and then evaluated the impact on predictors of new-onset hypertension when the new definition (130/80 mmHg) was used instead of the traditional definition (140/90 mmHg) during a mean follow-up period of 3.9 years. We found that, after multivariable analysis, older age, male sex, high SBP, high DBP, high heart rate, high BMI, high fasting glucose, and high triglycerides were significantly associated with new-onset hypertension in the new definition population. When comparing the two study populations, older age, high SBP and DBP, high heart rate, high BMI, high fasting glucose, and high triglycerides were significant predictors of new-onset hypertension in both study populations; however, male sex was a significant predictor of new-onset hypertension only in the new definition population, and low cholesterol and low eGFR were significant predictors of new-onset hypertension only in the traditional definition population.

The first important finding of this study is that male sex was a significant predictor of new-onset hypertension only in the new definition population. Sex has been reported to be a key factor in the development of hypertension. A previous study demonstrated that men had a higher prevalence of hypertension than women among subjects aged 18–59 years, but that men had a lower prevalence of hypertension than women in subjects older than 60 years [21]. A higher prevalence of hypertension in men compared to age-matched, pre-menopausal women may be caused by the effect of estrogen. Estrogen affects mediators of blood pressure, such as vascular tone, increasing nitric oxide bioavailability, inhibiting vascular remodeling, and mediating components of the renin–angiotensin–aldosterone system [22,23] and, therefore, estrogen may have a significant blood pressure-lowering effect. In this study, baseline SBP and DBP and longitudinal changes in SBP and DBP were higher in the male than in the female participants in both the new and traditional definition populations. The mean ages of our study subjects were 49 ± 10 and 50 ± 10 years in the new and traditional definition populations, respectively. This relatively young age (<60 years) may explain the higher baseline SBP and DBP and longitudinal changes in SBP and DBP in the male than in the female participants in both groups. In addition, the longitudinal changes in SBP in the male and female participants in the new definition population were 9.8 ± 12.4 and 7.7 ± 12.6 mmHg, respectively, so the mean difference in the longitudinal change in SBP between the male and female participants was 2.1 mmHg. The longitudinal changes in DBP in the male and female participants in the new definition population were 3.5 ± 8.2 and 2.6 ± 8.1 mmHg, respectively, so the mean difference in the longitudinal change in DBP between the male and female participants was 0.9 mmHg. Similarly, the longitudinal changes in SBP in the male and female participants in the traditional definition population were 9.0 ± 13.0 and 7.3 ± 13.1 mmHg, respectively, so the mean difference in the longitudinal change in SBP between the male and female participants was 1.7 mmHg. The longitudinal changes in DBP in the male and female participants in the traditional definition population were 2.4 ± 8.6 and 2.0 ± 8.6 mmHg, respectively, so the mean difference in the longitudinal change in DBP between the male and female participants was 0.4 mmHg. Hence, the mean differences in longitudinal changes in SBP and DBP between the male and female participants (2.1 vs. 1.7 mmHg and 0.9 vs. 0.4 mmHg, respectively) were numerically larger in the new definition population than in the traditional definition population. This may partially explain why male sex was a significant predictor of new-onset hypertension in the new definition population but not in the traditional definition population. 

The second important finding of this study is that high cholesterol was not a significant predictor of new-onset hypertension in either the new or traditional definition population. Previous studies evaluating the association between high cholesterol and new-onset hypertension have shown inconsistent results. He et al. investigated the association of lipid profiles with new-onset hypertension in a community-based non-hypertensive cohort of 1802 Chinese participants without lipid-lowering treatment, and found that higher triglycerides and lower low-density lipoprotein cholesterol increased the risk of new-onset hypertension, but that for total cholesterol, low-density lipoprotein cholesterol and non-high-density lipoprotein cholesterol, the risk of new-onset hypertension was increased only at normal concentrations. In contrast, hypercholesterolemia was negatively associated with new-onset hypertension [24]. Borghi et al. investigated possible interactions between serum cholesterol levels and the renin–angiotensin system on the development of stable hypertension in 66 young (age < 45 years) patients with high-normal blood pressure, and demonstrated that the presence of hypercholesterolemia could promote the development of stable hypertension through its interaction with the circulating renin–angiotensin system [25]. In our present large-scale study, high cholesterol was significantly associated with new-onset hypertension in both the new and traditional definition populations in univariable analysis. However, this association was attenuated after multivariable analysis. In the new definition group, total cholesterol became insignificant in multivariable analysis, and it was negatively associated in the traditional definition population after adjustments. The precise mechanism of this finding is unclear. In summary, we found that hypercholesterolemia was not significantly associated with new-onset hypertension in either the new or traditional definition group.

The third important finding of this study is that low eGFR was significantly associated with new-onset hypertension in the traditional definition population (*p* < 0.001), whereas the association was only borderline significant in the new definition population (*p* = 0.051). Hence, lowering the hypertensive diagnostic cut-off value may affect the association between low eGFR and new-onset hypertension. Impaired eGFR has frequently been reported to be a significant predictor of new-onset hypertension in previous studies [26,27]; however, the exact mechanism is unclear. Compared to the new definition population, the mean eGFR was numerically lower in the traditional definition population (111 ± 25 vs. 112 ± 25 mL/min/1.73 m^2^). The relatively lower eGFR may partially explain why a low eGFR was a significant predictor of new-onset hypertension only in the traditional definition population.

The fourth important finding of this study is that older age, high SBP and DBP, high heart rate, high BMI, high fasting glucose, and high triglycerides were significant predictors of new-onset hypertension in both study populations. Changing the diagnostic cut-off value for hypertension did not alter these associations. Many previous studies have consistently confirmed the significant associations between these clinical and laboratory parameters and new-onset hypertension [24,27,28,29,30,31]. 

Another important finding of this study is that follow-up SBP, DBP, and PP had different predictors in the new and traditional definition population. However, follow-up MAP had consistent predictors in both populations, including age, sex, DM, heart rate, BMI, fasting glucose, total cholesterol, and triglycerides. These results illustrate that these predictors are valid for both populations. Current guidelines have changed the diagnostic cut-off value from 140/90 mmHg to 130/80 mmHg [10,11]. Although the recommended guidelines for blood pressure can help to diagnose and treat hypertension, blood pressure is a continuous process that changes with age and other risk factors. Therefore, in the prevention of hypertension, in addition to analyzing the risk factors for new-onset hypertension under different definitions, it is also important to consider continuous changes in the risk factors for different blood pressure markers (such as follow-up SBP, DBP, PP, and MAP).

## 4. Study Limitations

There were several limitations to this study. First, our participants received surveys and examinations only at baseline and at a mean 3.9 years of follow-up, so the exact time of hypertension onset was unknown. Second, we had no data for physical activity, family history of hypertension, or dietary habits, so we could not analyze associations between these parameters and new-onset hypertension. Third, we used office blood pressure measurements to determine the values of SBP and DPB, so the white-coat effect could not be completely excluded. Finally, the follow-up duration was relatively short (mean 3.9 years). A longer follow-up period may improve the strength of the results. 

## 5. Conclusions

Our results demonstrated that older age, high SBP and DBP, high heart rate, high BMI, high fasting glucose, and high triglycerides were useful predictors of new-onset hypertension in both the new and traditional definition populations. However, male sex was a significant predictor of new-onset hypertension only in the new definition population, and low cholesterol and low eGFR were significant predictors of new-onset hypertension only in the traditional definition population. Hence, changing the diagnostic cut-off value for hypertension may have a significant impact on the association of some clinical and laboratory parameters with new-onset hypertension. 

## Figures and Tables

**Table 1:** Comparisons of baseline characteristics in the new definition population (hypertension was defined as systolic blood pressure ≥130 mmHg or diastolic blood pressure ≥80 mmHg).

Characteristics	Participants with New-Onset Hypertension (*n* = 3641)	Participants without New-Onset Hypertension (*n* = 13,431)	*p* Value	All Participants (*n* = 17,072)
Age (year)	53 ± 10	48 ± 10	<0.001	49 ± 10
Male sex (%)	39	24	<0.001	27
Smoking history (%)	28	20	<0.001	21
Diabetes mellitus (%)	5.3	2.7	<0.001	3.3
Systolic blood pressure (mmHg)	115 ± 9	106 ± 10	<0.001	108 ± 11
Diastolic blood pressure (mmHg)	71 ± 6	66 ± 7	<0.001	67 ± 7
Heart rate (beat/min)	70 ± 9	69 ± 9	0.031	69 ± 9
Body mass index (kg/m^2^)	24.5 ± 3.3	22.9 ± 3.2	<0.001	23.3 ± 3.3
Fasting glucose (g/dL)	97.7 ± 23.7	92.4 ± 15.1	<0.001	94 ± 17
Total cholesterol (mg/dL)	198 ± 35	193 ± 35	<0.001	194 ± 35
Triglycerides (mg/dL)	121 ± 93	97 ± 66	<0.001	102 ± 74
eGFR (mL/min/1.73 m^2^)	108 ± 26	114 ± 25	<0.001	112 ± 25

Here, eGFR is estimated glomerular filtration rate.

**Table 2:** Comparisons of baseline characteristics in the traditional definition population (hypertension was defined as systolic blood pressure ≥140 mmHg or diastolic blood pressure ≥90 mmHg).

Characteristics	Participants with New-Onset Hypertension (*n* = 3002)	Participants without New-Onset Hypertension (*n* = 18,291)	*p* Value	All Participants (*n* = 21,293)
Age (year)	55 ± 9	49 ± 10	<0.001	50 ± 10
Male sex (%)	43	30	<0.001	32
Smoking history (%)	18	13	<0.001	14
Diabetes mellitus (%)	6.6	3.0	<0.001	3.5
Systolic blood pressure (mmHg)	124 ± 10	110 ± 12	<0.001	112 ± 13
Diastolic blood pressure (mmHg)	76 ± 8	69 ± 9	<0.001	70 ± 9
Heart rate (beat/min)	70 ± 9	69 ± 9	0.001	69.± 9
Body mass index (kg/m^2^)	24.9 ± 3.4	23.4 ± 3.3	<0.001	23.6 ± 3.4
Fasting glucose (g/dL)	99 ± 25	94 ± 17	<0.001	94 ± 19
Total cholesterol (mg/dL)	200 ± 36	194 ± 35	<0.001	195 ± 35
Triglycerides (mg/dL)	129 ± 98	104 ± 76	<0.001	108 ± 80
eGFR (mL/min/1.73 m^2^)	105 ± 25	112 ± 25	<0.001	111 ± 25

Here, eGFR is estimated glomerular filtration rate.

**Table 3:** Determinants of new-onset hypertension using binary logistic analysis in the new definition population (hypertension was defined as systolic blood pressure ≥130 mmHg or diastolic blood pressure ≥80 mmHg).

Parameter	Univariable		Multivariable	
	OR (95% CI)	*p*	OR (95% CI)	*p*
Age (per 1 year)	1.051 (1.047–1.055)	<0.001	1.035 (1.030–1.039)	<0.001
Male (vs. female)	2.044 (1.892–2.209)	<0.001	1.332 (1.194–1.486)	<0.001
Smoking history	1.558 (1.432–1.695)	<0.001	1.030 (0.920–1.154)	0.605
Diabetes mellitus	2.015 (1.686–2.409)	<0.001	0.863 (0.686–1.085)	0.208
Systolic blood pressure (per 1 mmHg)	1.103 (1.098–1.107)	<0.001	1.067 (1.062–1.073)	<0.001
Diastolic blood pressure (per 1 mmHg)	1.122 (1.115–1.129)	<0.001	1.048 (1.040–1.056)	<0.001
Heart rate (per 1 beat/min)	1.005 (1.000–1.009)	0.031	1.007 (1.002–1.012)	0.004
Body mass index (per 1 kg/m^2^)	1.155 (1.142–1.168)	<0.001	1.091 (1.077–1.106)	<0.001
Fasting glucose (per 1 g/dL)	1.015 (1.013–1.017)	<0.001	1.004 (1.001–1.006)	0.002
Total cholesterol (per 1 mg/dL)	1.004 (1.003–1.005)	<0.001	0.999 (0.998–1.000)	0.058
Triglyceride (per 1 mg/dL)	1.004 (1.003–1.005)	<0.001	1.001 (1.000–1.001)	0.004
eGFR (per 1 mL/min/1.73 m^2^)	0.990 (0.988–0.991)	<0.001	0.998 (0.996–1.000)	0.051

Values expressed as odds ratio (OR) and 95% confidence interval (CI). Here, eGFR is the estimated glomerular filtration rate.

**Table 4:** Determinants of new-onset hypertension using binary logistic analysis in the traditional definition population (hypertension was defined as systolic blood pressure ≥140 mmHg or diastolic blood pressure ≥90 mmHg).

Parameter	Univariable		Multivariable	
	OR (95% CI)	*p*	OR (95% CI)	*p*
Age (per 1 year)	1.064 (1.060–1.069)	<0.001	1.038 (1.032–1.043)	<0.001
Male (vs. female)	1.750 (1.617–1.894)	<0.001	0.948 (0.845–1.064)	0.364
Smoking history	1.441 (1.322–1.570)	<0.001	1.057 (0.939–1.190)	0.360
Diabetes mellitus	2.304 (1.948–2.724)	<0.001	1.195 (0.962–1.482)	0.107
Systolic blood pressure (per 1 mmHg)	1.109 (1.104–1.113)	<0.001	1.078 (1.072–1.084)	<0.001
Diastolic blood pressure (per 1 mmHg)	1.114 (1.108–1.120)	<0.001	1.039 (1.031–1.046)	<0.001
Heart rate (per 1 beat/min)	1.007 (1.003–1.012)	0.001	1.005 (1.000–1.010)	0.032
Body mass index (per 1 kg/m^2^)	1.139 (1.126–1.151)	<0.001	1.072 (1.058–1.087)	<0.001
Fasting glucose (per 1 g/dL)	1.012 (1.010–1.014)	<0.001	1.003 (1.000–1.005)	0.020
Total cholesterol (per 1 mg/dL)	1.004 (1.003–1.005)	<0.001	0.998 (0.997–0.999)	0.004
Triglyceride (per 1 mg/dL)	1.003 (1.003–1.004)	<0.001	1.001 (1.000–1.001)	0.001
eGFR (per 1 mL/min/1.73 m^2^)	0.987 (0.985–0.988)	<0.001	0.995 (0.993–0.997)	<0.001

Values expressed as odds ratio (OR) and 95% confidence interval (CI). Here, eGFR is the estimated glomerular filtration rate.

## Data Availability

The data that support the findings of this study are available from Taiwan Biobank, but restrictions apply to the availability of these data, which were used under license for the current study, and so are not publicly available. Data are, however, available from the authors upon reasonable request and with permission of Taiwan Biobank.

## Supplementary Materials

The following supporting information can be downloaded at: https://www.mdpi.com/article/10.3390/ijerph192416525/s1, Table S1: Determinants of follow-up systolic blood pressure using linear regression analysis in new definition population (hypertension was defined as systolic blood pressure ≥130 mmHg or diastolic blood pressure ≥80 mmHg); Table S2: Determinants of follow-up systolic blood pressure using linear regression analysis in traditional definition population (hypertension was defined as systolic blood pressure ≥140 mmHg or diastolic blood pressure ≥90 mmHg); Table S3: Determinants of follow-up diastolic blood pressure using linear regression analysis in new definition population (hypertension was defined as systolic blood pressure ≥130 mmHg or diastolic blood pressure ≥80 mmHg); Table S4: Determinants of follow-up diastolic blood pressure using linear regression analysis in traditional definition population (hypertension was defined as systolic blood pressure ≥140 mmHg or diastolic blood pressure ≥90 mmHg); Table S5: Determinants of follow-up pulse pressure using linear regression analysis in new definition population (hypertension was defined as systolic blood pressure ≥130 mmHg or diastolic blood pressure ≥80 mmHg); Table S6. Determinants of follow-up pulse pressure using linear regression analysis in traditional definition population (hypertension was defined as systolic blood pressure ≥140 mmHg or diastolic blood pressure ≥90 mmHg); Table S7: Determinants of follow-up mean arterial pressure using linear regression analysis in new definition population (hypertension was defined as systolic blood pressure ≥130 mmHg or diastolic blood pressure ≥80 mmHg); Table S8: Determinants of follow-up mean arterial pressure using linear regression analysis in traditional definition population (hypertension was defined as systolic blood pressure ≥140 mmHg or diastolic blood pressure ≥90 mmHg).
