# Supplementary material for: Prognostic Factors of New-Onset Hypertension in New and Traditional Hypertension Definition in a Large Taiwanese Population Follow-up Study
Source: Int J Environ Res Public Health. 2022 Dec 9;19(24):16525. doi: 10.3390/ijerph192416525 (PMC9779332; doi:10.3390/ijerph192416525)
Supplement: Supplementary file 1 [file ijerph-19-16525-s001.zip › ijerph-2050302-supplementary.pdf]

Table S1. Determinants of follow-up systolic blood pressure using linear regression analysis in new definition population (hypertension was defined as systolic blood pressure ≥ 130 mmHg or diastolic blood pressure ≥ 80 mmHg)

Parameter	Univariable		Multivariable	
	Unstandardized coefficient β (95% CI)	*p*	OR (95% CI)	*p*
Age (per 1 year)	0.449 (0.429, 0.469)	< 0.001	0.420 (0.399, 0.441)	< 0.001
Male (*vs.* female)	6.593 (6.115, 7.072)	< 0.001	5.719 (5.174, 6.264)	< 0.001
Smoking history	3.037 (2.508, 3.556)	< 0.001	-1.034 (-1.599, -0.469)	< 0.001
Diabetes mellitus	6.787 (5.563, 8.011)	< 0.001	-0.825 (-2.085, 0.435)	0.199
Heart rate (per 1 beat/min)	-0.019 (-0.044, 0.006)	0.145	—	—
Body mass index (per 1 kg/m^2)	1.185 (1.120, 1.249)	< 0.001	0.930 (0.866, 0.994)	< 0.001
Fasting glucose (per 1 g/dl)	0.137 (0.124, 0.149)	< 0.001	0.035 (0.021, 0.048)	< 0.001
Total cholesterol (per 1 mg/dl)	0.049 (0.043, 0.055)	< 0.001	0.009 (0.003, 0.015)	0.002
Triglyceride (per 1 mg/dl)	0.035 (0.032, 0.038)	<0.001	0.009 (0.006, 0.012)	< 0.001
eGFR (per 1 ml/ min/1.73 m^2)	-0.076 (-0.085, -0.068)	< 0.001	0.010 (0.002, 0.019)	0.02

Values expressed as odds ratio (OR) and 95% confidence interval (CI). eGFR, estimated glomerular filtration rate.

Table S2. Determinants of follow-up systolic blood pressure using linear regression analysis in traditional definition population (hypertension was defined as systolic blood pressure ≥ 140 mmHg or diastolic blood pressure ≥ 90 mmHg)

Parameter	Univariable		Multivariable	
	Unstandardized coefficient β (95% CI)	*p*	OR (95% CI)	*p*
Age (per 1 year)	0.508 (0.488, 0.528)	< 0.001	0.483 (0.463, 0.503)	< 0.001
Male (*vs.* female)	7.687 (7.235, 8.139)	< 0.001	6.456 (5.947, 6.983)	< 0.001
Smoking history	3.929 (3.423, 4.434)	< 0.001	-1.027 (-1.567, -0.487)	< 0.001
Diabetes mellitus	6.496 (5.322, 7.669)	< 0.001	-1.442 (-2.636, -0.257)	0.017
Heart rate (per 1 beat/min)	0.012 (-0.013, 0.036)	0.348	—	—
Body mass index (per 1 kg/m^2)	1.303 (1.242, 1.364)	< 0.001	1.019 (0.959, 1.080)	< 0.001
Fasting glucose (per 1 g/dl)	0.142 (0.131, 0.154)	< 0.001	0.036 (0.024, 0.048)	< 0.001
Total cholesterol (per 1 mg/dl)	0.054 (0.048, 0.060)	< 0.001	0.012 (0.006, 0.018)	< 0.001
Triglyceride (per 1 mg/dl)	0.036 (0.034, 0.039)	<0.001	0.010 (0.007, 0.013)	< 0.001
eGFR (per 1 ml/ min/1.73 m^2)	-0.094 (-0.102, -0.085)	< 0.001	0.008 (-0.001, 0.016)	0.072

Values expressed as odds ratio (OR) and 95% confidence interval (CI). eGFR, estimated glomerular filtration rate.

Table S3. Determinants of follow-up diastolic blood pressure using linear regression analysis in new definition population (hypertension was defined as systolic blood pressure ≥ 130 mmHg or diastolic blood pressure ≥ 80 mmHg)

Parameter	Univariable		Multivariable	
	Unstandardized coefficient β (95% CI)	*p*	Unstandardized coefficient β (95% CI)	*p*
Age (per 1 year)	0.046 (0.033, 0.059)	< 0.001	0.036 (0.023, 0.049)	< 0.001
Male (*vs.* female)	5.039 (4.751, 5.326)	< 0.001	4.170 (3.824, 4.516)	< 0.001
Smoking history	3.224 (2.905, 3.544)	< 0.001	0.110 (-0.248, 0.468)	0.548
Diabetes mellitus	-0.116 (-0.864, 0.632)	0.76	—	—
Heart rate (per 1 beat/min)	0.079 (0.063, 0.094)	< 0.001	0.090 (0.076, 0.105)	< 0.001
Body mass index (per 1 kg/m^2)	0.732 (0.693, 0.771)	< 0.001	0.573 (0.532, 0.613)	< 0.001
Fasting glucose (per 1 g/dl)	0.039 (0.031, 0.046)	< 0.001	-0.009 (-0.016, -0.001)	< 0.001
Total cholesterol (per 1 mg/dl)	0.015 (0.011, 0.018)	< 0.001	0.008 (0.004, 0.012)	< 0.001
Triglyceride (per 1 mg/dl)	0.020 (0.018, 0.022)	<0.001	0.006 (0.005, 0.008)	< 0.001
eGFR (per 1 ml/ min/1.73 m^2)	-0.036 (-0.042, -0.031)	< 0.001	-0.005 (-0.011, 0.000)	0.055

Values expressed as odds ratio (OR) and 95% confidence interval (CI). eGFR, estimated glomerular filtration rate.

Table S4. Determinants of follow-up diastolic blood pressure using linear regression analysis in traditional definition population (hypertension was defined as systolic blood pressure ≥ 140 mmHg or diastolic blood pressure ≥ 90 mmHg)

Parameter	Univariable		Multivariable	
	Unstandardized coefficient β (95% CI)	*p*	Unstandardized coefficient β (95% CI)	*p*
Age (per 1 year)	0.060 (0.047, 0.073)	< 0.001	0.053 (0.040, 0.066)	< 0.001
Male (*vs.* female)	6.142 (5.871, 6.412)	< 0.001	5.061 (4.734, 5.389)	< 0.001
Smoking history	3.968 (3.662, 4.274)	< 0.001	0.088 (-0.253, 0.429)	0.614
Diabetes mellitus	-0.456 (-1.176, 0.263)	0.214	—	—
Heart rate (per 1 beat/min)	0.103 (0.088, 0.118)	< 0.001	0.109 (0.095, 0.123)	< 0.001
Body mass index (per 1 kg/m^2)	0.846 (0.808, 0.883)	< 0.001	0.644 (0.606, 0.682)	< 0.001
Fasting glucose (per 1 g/dl)	0.046 (0.039, 0.053)	< 0.001	-0.010 (-0.017, -0.003)	0.006
Total cholesterol (per 1 mg/dl)	0.018 (0.014, 0.022)	< 0.001	0.010 (0.006, 0.013)	< 0.001
Triglyceride (per 1 mg/dl)	0.022 (0.021, 0.024)	<0.001	0.007 (0.005, 0.008)	< 0.001
eGFR (per 1 ml/ min/1.73 m^2)	-0.047 (-0.052, -0.041)	< 0.001	-0.006 (-0.011, 0.000)	0.041

Values expressed as odds ratio (OR) and 95% confidence interval (CI). eGFR, estimated glomerular filtration rate.

Table S5. Determinants of follow-up pulse pressure using linear regression analysis in new definition population (hypertension was defined as systolic blood pressure ≥ 130 mmHg or diastolic blood pressure ≥ 80 mmHg)

Parameter	Univariable		Multivariable	
	Unstandardized coefficient β (95% CI)	*p*	OR (95% CI)	*p*
Age (per 1 year)	0.403 (0.390, 0.417)	< 0.001	0.385 (0.370, 0.399)	< 0.001
Male (*vs.* female)	1.555 (1.212, 1.897)	< 0.001	1.032 (0.699, 1.365)	< 0.001
Smoking history	-0.187 (-0.560, 0.186)	0.325	—	—
Diabetes mellitus	6.904 (6.047, 7.760)	< 0.001	1.755 (0.868, 2.642)	< 0.001
Heart rate (per 1 beat/min)	-0.097 (-0.115, -0.080)	< 0.001	-0.072 (-0.088, -0.056)	< 0.001
Body mass index (per 1 kg/m^2)	0.453 (0.406, 0.499)	< 0.001	0.348 (0.303, 0.393)	< 0.001
Fasting glucose (per 1 g/dl)	0.098 (0.089, 0.107)	< 0.001	0.031 (0.021, 0.040)	< 0.001
Total cholesterol (per 1 mg/dl)	0.034 (0.030, 0.039)	< 0.001	0.003 (-0.001, 0.008)	0.115
Triglyceride (per 1 mg/dl)	0.015 (0.013, 0.017)	<0.001	0.002 (0.000, 0.005)	0.019
eGFR (per 1 ml/ min/1.73 m^2)	-0.040 (-0.046, -0.034)	< 0.001	0.015 (0.009, 0.021)	0.903

Values expressed as odds ratio (OR) and 95% confidence interval (CI). eGFR, estimated glomerular filtration rate.

Table S6. Determinants of follow-up pulse pressure using linear regression analysis in traditional definition population (hypertension was defined as systolic blood pressure ≥ 140 mmHg or diastolic blood pressure ≥ 90 mmHg)

Parameter	Univariable		Multivariable	
	Unstandardized coefficient β (95% CI)	*p*	OR (95% CI)	*p*
Age (per 1 year)	0.448 (0.435, 0.461)	< 0.001	0.429 (0.416, 0.443)	< 0.001
Male (*vs.* female)	1.546 (1.232, 1.859)	< 0.001	0.935 (0.632, 1.239)	< 0.001
Smoking history	-0.039 (-0.384, 0.305)	0.823	—	—
Diabetes mellitus	6.952 (6.160, 7.744)	< 0.001	1.724 (0.918, 2.529)	< 0.001
Heart rate (per 1 beat/min)	-0.091 (-0.108, 0.075)	< 0.001	-0.07 (-0.085, -0.055)	< 0.001
Body mass index (per 1 kg/m^2)	0.457 (0.415, 0.500)	< 0.001	0.365 (0.325, 0.406)	< 0.001
Fasting glucose (per 1 g/dl)	0.096 (0.088, 0.104)	< 0.001	0.030 (0.022, 0.038)	< 0.001
Total cholesterol (per 1 mg/dl)	0.036 (0.032, 0.016)	< 0.001	0.004 (0.001, 0.008)	0.027
Triglyceride (per 1 mg/dl)	0.014 (0.012, 0.016)	<0.001	0.003 (0.001, 0.005)	0.003
eGFR (per 1 ml/ min/1.73 m^2)	-0.047 (-0.053, -0.041)	< 0.001	0.012 (0.006, 0.018)	< 0.001

Values expressed as odds ratio (OR) and 95% confidence interval (CI). eGFR, estimated glomerular filtration rate.

Table S7. Determinants of follow-up mean arterial pressure using linear regression analysis in new definition population (hypertension was defined as systolic blood pressure ≥ 130 mmHg or diastolic blood pressure ≥ 80 mmHg)

Parameter	Univariable		Multivariable	
	Unstandardized coefficient β (95% CI)	*p*	OR (95% CI)	*p*
Age (per 1 year)	0.180 (0.166, 0.195)	< 0.001	0.167 (0.152, 0.182)	< 0.001
Male (*vs.* female)	5.557 (5.232, 5.882)	< 0.001	4.687 (4.304, 5.069)	< 0.001
Smoking history	3.162 (2.801, 3.523)	< 0.001	-0.261 (-0.656, 0.135)	0.196
Diabetes mellitus	2.185 (1.343, 3.027)	< 0.001	-2.047 (-2.930, -1.165)	< 0.001
Heart rate (per 1 beat/min)	0.046 (0.029, 0.063)	< 0.001	0.068 (0.052, 0.084)	< 0.001
Body mass index (per 1 kg/m^2)	0.883 (0.839, 0.927)	< 0.001	0.693 (0.648, 0.738)	< 0.001
Fasting glucose (per 1 g/dl)	0.071 (0.063, 0.080)	< 0.001	0.013 (0.004, 0.023)	0.004
Total cholesterol (per 1 mg/dl)	0.026 (0.022, 0.030)	< 0.001	0.007 (0.003, 0.012)	0.001
Triglyceride (per 1 mg/dl)	0.025 (0.023, 0.027)	<0.001	0.007 (0.005, 0.009)	< 0.001
eGFR (per 1 ml/ min/1.73 m^2)	-0.050 (-0.056, -0.044)	< 0.001	0.000 (-0.006, 0.006)	0.903

Values expressed as odds ratio (OR) and 95% confidence interval (CI). eGFR, estimated glomerular filtration rate.

Table S8. Determinants of follow-up mean arterial pressure using linear regression analysis in traditional definition population (hypertension was defined as systolic blood pressure ≥ 140 mmHg or diastolic blood pressure ≥ 90 mmHg)

Parameter	Univariable		Multivariable	
	Unstandardized coefficient β (95% CI)	*p*	OR (95% CI)	*p*
Age (per 1 year)	0.209 (0.195, 0.224)	< 0.001	0.201 (0.187, 0.215)	< 0.001
Male (*vs.* female)	6.657 (6.348, 6.965)	< 0.001	5.529 (5.164, 5.894)	< 0.001
Smoking history	3.955 (3.607, 4.303)	< 0.001	-0.271 (-0.650, 0.109)	0.163
Diabetes mellitus	1.861 (1.046, 2.676)	< 0.001	-2.689 (-3.524, -1.854)	< 0.001
Heart rate (per 1 beat/min)	0.072 (0.055, 0.089)	< 0.001	0.088 (0.073, 0.104)	< 0.001
Body mass index (per 1 kg/m^2)	0.998 (0.956, 1.040)	< 0.001	0.770 (0.728, 0.813)	< 0.001
Fasting glucose (per 1 g/dl)	0.078 (0.070, 0.086)	< 0.001	0.014 (0.006, 0.023)	0.001
Total cholesterol (per 1 mg/dl)	0.030 (0.026, 0.034)	< 0.001	0.009 (0.005, 0.013)	< 0.001
Triglyceride (per 1 mg/dl)	0.027 (0.025, 0.029)	<0.001	0.007 (0.006, 0.009)	< 0.001
eGFR (per 1 ml/ min/1.73 m^2)	-0.062 (-0.068, -0.056)	< 0.001	-0.001 (-0.007, 0.004)	0.629

Values expressed as odds ratio (OR) and 95% confidence interval (CI). eGFR, estimated glomerular filtration rate.
